# Correction: Lin et al. Grounding the Body Improves Sleep Quality in Patients with Mild Alzheimer’s Disease: A Pilot Study. *Healthcare* 2022, *10*, 581

**DOI:** 10.3390/healthcare10060988

**Published:** 2022-05-26

**Authors:** Chien-Hung Lin, Shih-Ting Tseng, Yao-Chung Chuang, Chun-En Kuo, Nai-Ching Chen

**Affiliations:** 1 Department of Chinese Medicine, Kaohsiung Chang Gung Memorial Hospital, College of Medicine, Chang Gung University, Kaohsiung 833, Taiwan; b9505027@cgmh.org.tw (C.-H.L.); b9705017@cgmh.org.tw (S.-T.T.); 2 Department of Neurology, Kaohsiung Chang Gung Memorial Hospital, College of Medicine, Chang Gung University, Kaohsiung 833, Taiwan; ycchuang@ms87.url.com.tw; 3 School of Chinese Medicine for Post Baccalaureate I-Shou University, No. 1, Sec. 1, Syuecheng Rd., Dashu District, Kaohsiung 84001, Taiwan; 4 Department of Leisure and Sports Management, Cheng Shiu University, No. 840, Chengcing Rd., Niaosong Dist., Kaohsiung 83347, Taiwan

## Text Correction

The authors would like to make the following corrections to the published paper [1]. Replacing the Institutional Review Board number 2019011136B0 with 201901136B0 in Section 2.1. Ethics Approval and Institutional Review Board Statement.

The authors apologize for any inconvenience caused and state that the scientific conclusions are unaffected. This correction was approved by the Academic Editor. The original publication has also been updated.
